# PRC1 suppresses a female gene regulatory network to ensure testicular differentiation

**DOI:** 10.1038/s41419-023-05996-6

**Published:** 2023-08-04

**Authors:** So Maezawa, Masashi Yukawa, Kazuteru Hasegawa, Ryo Sugiyama, Mizuho Iizuka, Mengwen Hu, Akihiko Sakashita, Miguel Vidal, Haruhiko Koseki, Artem Barski, Tony DeFalco, Satoshi H. Namekawa

**Affiliations:** 1 Reproductive Sciences Center, Division of Developmental Biology, Perinatal Institute, Cincinnati Children’s Hospital Medical Center, Cincinnati, Ohio 45229 USA; 2 Department of Pediatrics, University of Cincinnati College of Medicine, Cincinnati, OH 45229 USA; 3 Department of Animal Science and Biotechnology, School of Veterinary Medicine, Azabu University, Sagamihara, Kanagawa 252-5201 Japan; 4 Department of Applied Biological Science, Faculty of Science and Technology, Tokyo University of Science, Chiba, 278-8510 Japan; 5 Division of Allergy and Immunology, Division of Human Genetics, Cincinnati Children’s Hospital Medical Center, Cincinnati, OH 45229 USA; 6 Department of Microbiology and Molecular Genetics, University of California, Davis, CA 95616 USA; 7 Centro de Investigaciones Biológicas Margarita Salas, Department of Cellular and Molecular Biology, Madrid, 28040 Spain; 8 Developmental Genetics Laboratory, RIKEN Center for Allergy and Immunology, Yokohama, Kanagawa Japan; 9 Present Address: Department of Chemical Pathology, The Chinese University of Hong Kong, Prince of Wales Hospital, Sha Tin, New Territories, Hong Kong; 10 Present Address: Department of Molecular Biology, Keio University School of Medicine, Tokyo, 160-8582 Japan

**Keywords:** Organogenesis, Epigenetic memory

## Abstract

Gonadal sex determination and differentiation are controlled by somatic support cells of testes (Sertoli cells) and ovaries (granulosa cells). In testes, the epigenetic mechanism that maintains chromatin states responsible for suppressing female sexual differentiation remains unclear. Here, we show that Polycomb repressive complex 1 (PRC1) suppresses a female gene regulatory network in postnatal Sertoli cells. We genetically disrupted PRC1 function in embryonic Sertoli cells after sex determination, and we found that PRC1-depleted postnatal Sertoli cells exhibited defective proliferation and cell death, leading to the degeneration of adult testes. In adult Sertoli cells, PRC1 suppressed specific genes required for granulosa cells, thereby inactivating the female gene regulatory network. Chromatin regions associated with female-specific genes were marked by Polycomb-mediated repressive modifications: PRC1-mediated H2AK119ub and PRC2-mediated H3K27me3. Taken together, this study identifies a critical Polycomb-based mechanism that suppresses ovarian differentiation and maintains Sertoli cell fate in adult testes.

## Introduction

In mammalian embryos, gonadal sex determination takes place in bipotential somatic cell precursors of male Sertoli cells and female granulosa cells [1–3]. Sertoli cells are the first somatic cells to differentiate in the XY gonad. In testes, Sertoli cells function as a regulatory hub for both differentiation and survival of germ cells, thereby determining male sexual fate [4]. The mechanisms maintaining the male cellular identity of Sertoli cells are fundamental for adult testicular functions, including spermatogenesis and hormone production.

At the time of sex determination in embryos, commitment to the male fate is triggered by the expression of the Y-linked *Sry* gene; subsequently, the female fate is suppressed [1–3, 5]. Distinct gene regulatory networks promote the male or female fate and are regulated by strong feedback loops that antagonize each other, canalizing one fate apart from the other [3]. Sexual fate is interchangeable even after the initial commitment to Sertoli cells or granulosa cells with the removal of specific, critical transcription factors. DMRT1 is the male-determining transcription factor [6, 7], and the loss of *Dmrt1* in Sertoli cells leads to the derepression of *Foxl2*, a master regulator of granulosa cell fate, and leads to transdifferentiation of cell fate from Sertoli to granulosa cells [8, 9]. Conversely, the loss of *Foxl2* in granulosa cells leads to derepression of the male gene network and transdifferentiation of cell fate from granulosa to Sertoli cells [10]. These findings, together with follow-up studies [11–15], suggest that active repression of the alternate sexual fate is important for both testicular and ovarian function, even in adult life. Of note, during sexual fate reprogramming from females to males, DMRT1 acts as a pioneer factor to open chromatin to allow the binding of SOX9 [16], an evolutionarily conserved factor for sex determination that directs the male pathway downstream of *Sry* [17].

Epigenetic silencing mechanisms serve as molecular switches for the sex determination of bipotential somatic cell precursors. The deletion of a Polycomb gene *Cbx2* results in XY male-to-female sex reversal [18], which is mediated through the derepression of genes required for the female fate [19]. Additionally, demethylation of H3K9 methylation at the *Sry* gene locus triggers *Sry* expression and the male sexual fate [20]. Although these studies highlight the importance of epigenetic mechanisms for initial sex determination, the epigenetic mechanisms by which male cellular identity is maintained through cell divisions and the proliferation of Sertoli cells remain to be determined.

Polycomb proteins suppress non-lineage-specific genes and define the cellular identities of each lineage in stem cells and in development [21–23]. Mammalian Polycomb proteins comprise two major complexes— Polycomb repressive complex 1 (PRC1) and PRC2—that mediate repressive histone marks, monoubiquitination of H2A at lysine 119 (H2AK119ub), and trimethylation of H3 at lysine 27 (H3K27me3), respectively [24]. Although earlier studies proposed that PRC2 functions upstream of PRC1 [25, 26], later studies revealed that variant PRC1 complexes function upstream of PRC2 and lead to the deposition of H3K27me3 [27, 28]. In addition, PRC1-mediated H2AK119ub is essential to maintain Polycomb gene repression [29, 30].

In this study, to determine the function of Polycomb silencing in the maintenance of the male pathway, we generated loss-of-function mouse models of PRC1 in Sertoli cells after initial sex determination. We show that PRC1 is required for the proliferation of Sertoli cells, as well as the suppression of non-lineage-specific genes and the female gene regulatory network in Sertoli cells. Taken together, we identify a critical mechanism centered on PRC1 that maintains male fate in adult testes.

## Results

### PRC1 in Sertoli cells is required for the maintenance of spermatogenesis

**Fig. 1:**
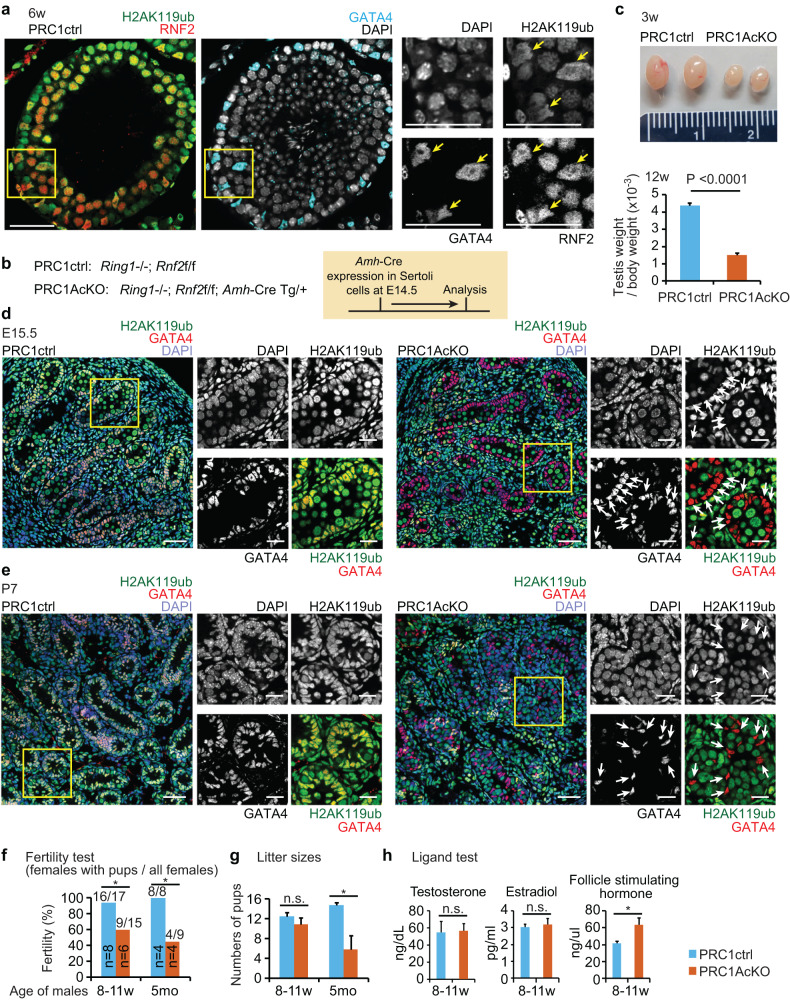
Deletion of PRC1 in Sertoli cells. **a** RNF2 and RNF2-mediated H2AK119ub localized at GATA4^+^ Sertoli cells (yellow arrows) in a testicular section at 6 weeks of age. Regions marked by yellow squares are magnified in the right panels. Bars in the large panels: 50 μm. Bars in the magnified panels: 20 μm. **b** Genotypes used in the experiments and schematic of experimental design. **c** (Top) Photographs of testes at 3 weeks of age. Measurement scale in the panel: 2 cm. (Bottom) Testicular weight/body weight ratio (× 10^-3^) at 12 weeks of age. *P* < 0.0001, unpaired t-test. **d, e** Localization of H2AK119ub and GATA4 in PRC1ctrl and PRC1AcKO at embryonic day 15.5 (**d**) and 1 week of age (**e**). Regions marked by yellow squares are magnified in the right panels. Bars in the large panels: 50 μm. Bars in the magnified panels: 20 μm. H2AK119ub- Sertoli cells in mutants are shown with white arrows. **f** The fertility of PRC1AcKO males at 8–11 weeks and 5 months of age was tested via crosses with CD1 wild-type females. Numbers of males tested are shown within the bars, and numbers of females with pups and all females are shown above the bars. *P < 0.05, Fisher’s exact test. **g** Litter sizes of breeding tests at 8-11 weeks (8-11 w) of age and 5 months (5 mo) of age. PRC1ctrl (8–11 w): 13, 13, 9, 9, 11, 8, 14, 11, 12, 13, 15, 0, 16, 16, 19, 13, and 8 pups from 17 female mice. PRC1AcKO (8-11 w): 12, 12, 9, 0, 14, 1, 0, 0, 2, 0, 15, 9, 12, 0, and 0 pups from 15 female mice. PRC1ctrl (5 mo): 12, 14, 18, 14, 15, 15, 15, and 15 pups from 8 female mice. PRC1AcKO (5 mo): 2, 5, 0, 0, 0, 14, 2, 0, and 0 pups from 9 female mice. *P < 0.05, Welch’s t-test. **h** Hormone tests (serum) at 8-11 weeks of age. *P < 0.05, Welch’s t-test. n.s., not significant. Data are presented as mean values ± SEM. Three independent mice were examined.

RNF2 (also known as RING1B), a catalytic subunit of PRC1, was highly expressed in Sertoli cells, as indicated by co-localization with the Sertoli cell marker GATA4, and the RNF2-mediated mark, H2AK119ub, was abundant in Sertoli cells (Fig. 1a, Supplementary Fig. 1), which suggest PRC1 functions in these cells. To determine the function of PRC1, we generated a PRC1 loss-of-function mouse model by removing two redundant catalytic subunits that mediate H2AK119ub deposition: RNF2 and RING1 (also known as RING1A) [31]. We generated a conditional deletion of *Rnf2* (*Rnf2*cKO) using *Amh*-Cre, which is expressed specifically in Sertoli cells starting at embryonic day 14.5 (E14.5) [32], in a background of *Ring1*-knockout (KO) mice (*Amh*-Cre; *Rnf2*cKO; *Ring1*-KO: termed PRC1^*Amh*-Cre^cKO: PRC1AcKO). Although RNF2 appears to be the most active component in the heterodimeric E3 ligases of PRC1, the RNF2 paralog RING1 can partially compensate for the loss of RNF2 [31]. Therefore, we made a conditional deletion of *Rnf2* in a background of *Ring1*-KO mice, which on their own are viable and do not have fertility defects [33]. This strategy enabled us to define the function of RNF2 without compensation from RING1 while also representing a "complete" loss-of-function of PRC1 as shown in testicular germ cells [34] and in other biological contexts [31, 35–37]. Since PRC1 has various components, including CBX2 [38], this strategy allowed us to determine the global function of PRC1. At the same time, the use of *Amh*-Cre allowed us to test the function of PRC1 specifically after the completion of initial sex determination by E12.5.

PRC1AcKO adult males had smaller testes compared with littermate controls that harbored floxed alleles for *Rnf2* on a *Ring1*-KO background without *Amh*-Cre (termed PRC1 control: PRC1ctrl: Fig. 1b and c). We observed depletion of RNF2 and the RNF2-mediated mark, H2AK119ub, in a part of GATA4^+^ Sertoli cells in 6-week-old PRC1AcKO testes (Supplementary Fig. 2a). We further confirmed nearly 80% efficiency of *Amh*-Cre-mediated recombination based on the population of cells that lacked H2AK119ub in GATA4^+^ Sertoli cells of PRC1AcKO testes at E15.5, postnatal day 1 (P1), and P7 (Fig. 1d, e, Supplementary Fig. 2b, c). In 6-week-old PRC1AcKO mice, testicular tubules with H2AK119ub^+^ Sertoli cells (i.e., those that escaped Cre-mediated deletion) appeared to have normal morphology; however, we observed disorganization of testicular tubules that contained H2AK119ub^-^ Sertoli cells (i.e., those with confirmed Cre-mediated deletion) in a minority of testicular tubules (yellow arrows in Supplementary Fig. 2a). This mosaic pattern is presumably due to mosaic Cre-mediated recombination, as Sertoli cells that escaped Cre-mediated recombination might have largely repopulated the testes by the age of 6 weeks old (Supplementary Fig. 2b). In support of this notion, disorganization of testicular tubules was not initially apparent in young testes at P1 and P7, but it became apparent in 6-week-old testes. To further examine the disorganization of testicular tubules, we next tested whether the blood-testis barrier was disrupted in PRC1AcKO testes. However, the overall distribution of tight junction proteins CLDN3 and CLDN11 that comprise the blood-testis barrier [39, 40] did not change in 6-week-old PRC1AcKO testes (Supplementary Fig. 3). This data suggests that blood-testis barrier formation was not disrupted in the disorganized testicular tubules of PRC1AcKO mice.

Consistent with overall testicular phenotypes, PRC1AcKO males were subfertile: only 9 out of 15 wild-type females mated with 8-11-week-old PRC1AcKO males gave birth (Fig. 1f), though litter sizes were comparable to those from controls (Fig. 1g). Interestingly, fecundity decreased in aged PRC1AcKO males (5 months old), as litter sizes were smaller compared to controls (Fig. 1g), and only 4 out of 9 wild-type females mated with 5-month-old PRC1AcKO males gave birth (Fig. 1f). To further examine the phenotype, we measured the blood levels of three hormones critical for testicular homeostasis: testosterone, estradiol, and follicle-stimulating hormone (FSH). Although the levels of testosterone and estradiol were comparable between cKO and control mice, FSH was increased in PRC1AcKO mice (Fig. 1h). As FSH levels are regulated by a feedback mechanism involving Sertoli cells [41], we infer that the dysfunction of Sertoli cells and testicular degeneration caused increased FSH levels in an attempt to recover Sertoli cell function.

**Fig. 2:**
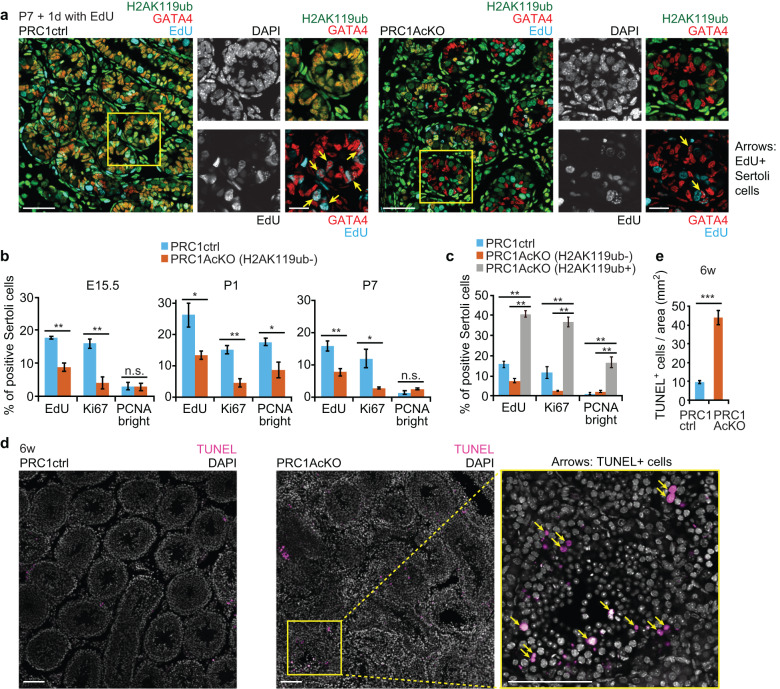
PRC1 is required for proliferation of Sertoli cells. **a** Testicular sections of the indicated genotypes 1 day following the injection of EdU into males at 1 week of age. The presence of EdU^+^ Sertoli cells (yellow arrows) was decreased in PRC1AcKO testes. Regions marked by yellow squares are magnified in the right panels. Bars in the large panels: 50 μm. Bars in the magnified panels: 20 μm. **b** Quantification of EdU^+^, Ki67^+^, and PCNA-bright Sertoli cells at E15.5, P1, and P7. Only H2AK119ub^-^ Sertoli cells were analyzed for PRC1AcKO testes. Data are presented as mean values ± SEM. **P < 0.01, *P < 0.05, unpaired t-tests. **c** Quantification of EdU^+^, Ki67^+^, and PCNA-bright Sertoli cells at P7. H2AK119ub^-^ Sertoli cells and H2AK119ub^+^ Sertoli cells were separately analyzed for PRC1AcKO testes. Data are presented as mean values ± SEM. **P < 0.01, unpaired t-tests. **d** TUNEL assay on testicular sections of the indicated genotypes at 6 weeks of age. Regions marked by yellow squares are magnified in the right panels. Bars in the large panels: 100 μm. Bars in the magnified panels: 100 μm. **e** Numbers of TUNEL^+^ cells per area (mm^2^) at 6 weeks of age. Data are presented as mean values ± SEM. ***P < 0.0001, Welch’s t-test. At least three independent mice were examined.

To determine the cause of testicular degeneration, we next examined the proliferation of Sertoli cells. In normal mouse development, Sertoli cells proliferate in fetal and neonatal testes until approximately 2 weeks after birth, and the number of Sertoli cells in adult testes determines both testis size and daily sperm production [42]. To examine proliferation, we first labeled actively proliferating cells using 5-ethynyl-2′-deoxyuridine (EdU) incorporation assays. EdU was administrated to P7 mice, and testicular sections were examined the following day (Supplementary Fig. 4a). While GATA4^+^ Sertoli cells were occasionally EdU^+^ (approximately 16-26%) in control testes at E15.5, P1, and P7, the frequency of EdU^+^ GATA4^+^ Sertoli cells was approximately 50% reduced in PRC1AcKO testes compared to controls (Fig. 2a, b, Supplementary Fig. 4b, c), which indicates decreased proliferation of PRC1AcKO Sertoli cells. Additional labeling of H2AK119ub confirmed the loss of PRC1 function in GATA4^+^ Sertoli cells from PRC1AcKO testes (Fig. 2a, Supplementary Fig. 4b, c).

Next, we independently confirmed the proliferation phenotype by using immunostaining for specific cell cycle markers. In P7 control testes, GATA4^+^ Sertoli cells occasionally co-expressed an active cell cycle marker, Ki67, and a replication protein, PCNA, which showed bright punctate signals within Sertoli nuclei (Supplementary Fig. 5), consistent with the active proliferation of Sertoli cells. However, in PRC1AcKO testes, the percentage of Ki67^+^ Sertoli cells was reduced at E15.5, P1, and P7, and the proportion of Sertoli cells with bright PCNA staining was decreased at P1 (Fig. 2b, Supplementary Fig. 5), suggesting decreased proliferation of PRC1AcKO Sertoli cells. From these results, we conclude that the loss of PRC1 decreases the proliferation of Sertoli cells. We further confirmed that H2AK119ub^+^ Sertoli cells (i.e., those that escaped Cre*-*mediated deletion) in PRC1AcKO testes were highly proliferative (high percentage of EdU^+^, Ki67^+^, and PCNA-bright cells) at P7 (Fig. 2c), indicating that the presence of PRC1 promoted the proliferation of Sertoli cells. This feature is in contrast with PRC1’s function in testicular germ cells, in which the loss of PRC1 does not affect proliferation but instead causes apoptotic cell death [34]. This difference suggests a unique function of PRC1 in Sertoli cells distinct from its function in germ cells.

Because we observed impaired spermatogenesis in PRC1AcKO testes, we next examined apoptotic cell death by TUNEL assay. In six-week-old PRC1AcKO testes, which show disorganization of minor populations of testicular tubules, TUNEL^+^ cells were locally increased in these disorganized tubules (Fig. 2d). On average, the frequency of apoptotic cells was increased by approximately 4-fold in PRC1AcKO testes compared to that of control testes (Fig. 2e). Therefore, increased cell death and reduced proliferation of PRC1-deleted Sertoli cells observed in PRC1AcKO testes may cause uneven proliferation of Sertoli cells, many of which are replenished after escape from *Cre*-mediated recombination at 6 weeks old, leading to the disorganization of minor populations of testicular tubules.

### In Sertoli cells, PRC1 suppresses genes required for granulosa cells

**Fig. 3:**
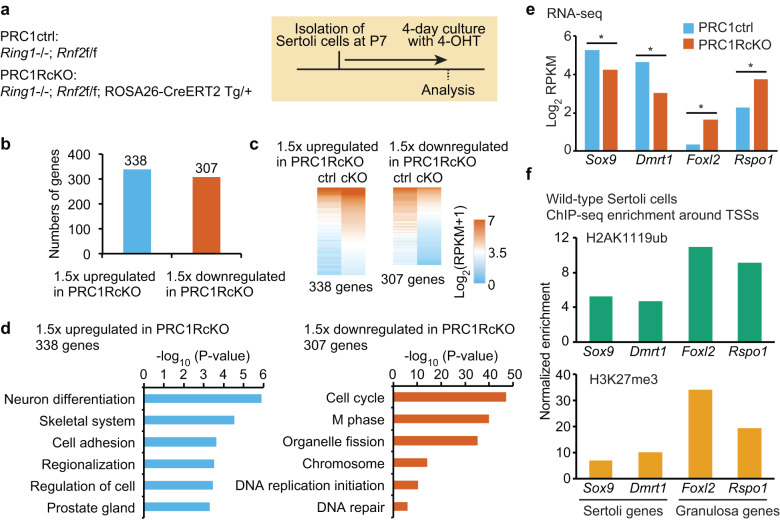
In Sertoli cells, PRC1 suppresses genes required for granulosa cells. **a** Genotypes used and schematic of experimental design. Sertoli cells were isolated from P7 testes and cultured for 4 days in the presence of 4-OHT prior to RNA-seq analyses. **b** Numbers of differentially expressed genes detected by RNA-seq (≥1.5-fold change, *P*_adj_ < 0.05) in Sertoli cells (Two biological replicates) between PRC1ctrl and PRC1RcKO. **c** Heatmaps showing gene expression patterns for upregulated (left) and downregulated (right) genes in Sertoli cells. **d** GO term analyses. **e** Expression levels for representative Sertoli and granulosa genes. **f** H2AK119ub and H3K27me3 ChIP-seq enrichment around the TSSs of representative Sertoli and granulosa genes.

We next sought to determine the genes regulated by PRC1 in Sertoli cells. In PRC1AcKO testes, some Sertoli cells escaped *Amh*-*Cre*-mediated recombination; thus, it was difficult to specifically isolate Sertoli cells that underwent PRC1 depletion. To precisely determine the function of PRC1 in gene regulation in Sertoli cells, we used an alternative strategy: we isolated Sertoli cells from a mouse line in which conditional deletion of PRC1 can be achieved by tamoxifen-inducible Cre-mediated recombination under the control of the endogenous ROSA26 promoter (ROSA26-CreERT2; *Rnf2*^floxed/floxed^; *Ring1*-KO: termed PRC1^ROSA26-CreERT2^cKO: PRC1RcKO). After isolating Sertoli cells from P7 testes, we cultured Sertoli cells for 4 days in the presence of 4-hydroxytamoxifen (4-OHT) and performed RNA-sequencing (RNA-seq: Fig. 3a, Supplementary Fig. 6a). As a control, we isolated Sertoli cells from control mice (*Rnf2*^floxed/floxed^; *Ring1*-KO) and cultured them in the same 4-OHT conditions. We confirmed specific depletion of RNF2 and H2AK119ub after a 4-day culture of PRC1RcKO Sertoli cells in the presence of 4-OHT (Supplementary Fig. 6b, Supplementary Material). We performed RNA-seq for two independent biological replicates and confirmed reproducibility between biological replicates (Supplementary Fig. 6c).

Our RNA-seq analyses demonstrated that 338 genes were upregulated in PRC1RcKO Sertoli cells as compared to controls, while 307 genes were downregulated in PRC1RcKO Sertoli cells (Fig. 3b and c, Supplementary File 1). Gene ontology (GO) analysis showed that upregulated genes were enriched for functions in neural differentiation, skeletal system, and cell adhesion (Fig. 3d). These categories suggest that PRC1 suppressed the expression of nonlineage-specific genes in Sertoli cells. On the other hand, GO analysis revealed that downregulated genes were enriched for functions in the cell cycle and M phase (Fig. 3d). This result is in accord with the reduced proliferation we found in PRC1AcKO Sertoli cells (Fig. 2).

Because we anticipated suppression of genes required for granulosa cells by PRC1 in Sertoli cells, we next investigated the expression level of key ovarian genes. In PRC1RcKO Sertoli cells, genes required for female sexual development were upregulated: these genes included *Rspo1*, an activator of the Wnt pathway [43], and *Foxl2*, a key transcription factor for granulosa cells [44] (Fig. 3e). We further confirmed that RSPO1 protein was ectopically expressed in PRC1RcKO Sertoli cells (Supplementary Fig. 6b, Supplementary Material). Importantly, these female genes suppress the male fate, and the loss of these genes leads to female-to-male sex reversal [44–46]. Consistent with the antagonistic function of these female genes with the male pathway, key sex determination genes for the male pathway were downregulated in PRC1RcKO Sertoli cells: these genes included *Sox9* and *Dmrt1* (Fig. 3e). Although we found gene expression changes and increased expression of RSPO1 protein in PRC1RcKO Sertoli cells, the immunostaining intensities of SOX9 were comparable between H2AK119ub^-^ and H2AK119ub^+^ Sertoli cells in PRC1AcKO testes (Supplementary Fig. 7). These results suggest that complete sex reversal did not take place in PRC1AcKO testes despite the ectopic expression of female genes.

To determine whether the suppression of female genes was directly regulated by PRC1, we performed chromatin immunoprecipitation sequencing (ChIP-seq) of PRC1-mediated H2AK119ub in isolated wild-type Sertoli cells. We further performed ChIP-seq of H3K27me3 in Sertoli cells because PRC2-mediated H3K27me3 is regulated by variant PRC1 and its mediated mark H2A119ub [27, 28]. We performed ChIP-seq for two independent biological replicates and confirmed reproducibility between biological replicates (Supplementary Fig. 6d, e). We confirmed the enrichment of H2AK119ub and H3K27me3 around transcription start sites (TSSs) of the *Hoxd* cluster, a classic Polycomb target (Supplementary Fig. 8), and the female genes *Rspo1* and *Foxl2* (Fig. 3f). Compared to the enrichment of H2AK119ub on these female genes, enrichment of H2AK119ub was relatively low on the TSSs of the male genes *Sox9* and *Dmrt1*. Therefore, we conclude that PRC1 directly binds and suppresses *Rspo1* and *Foxl2*.

**Fig. 4:**
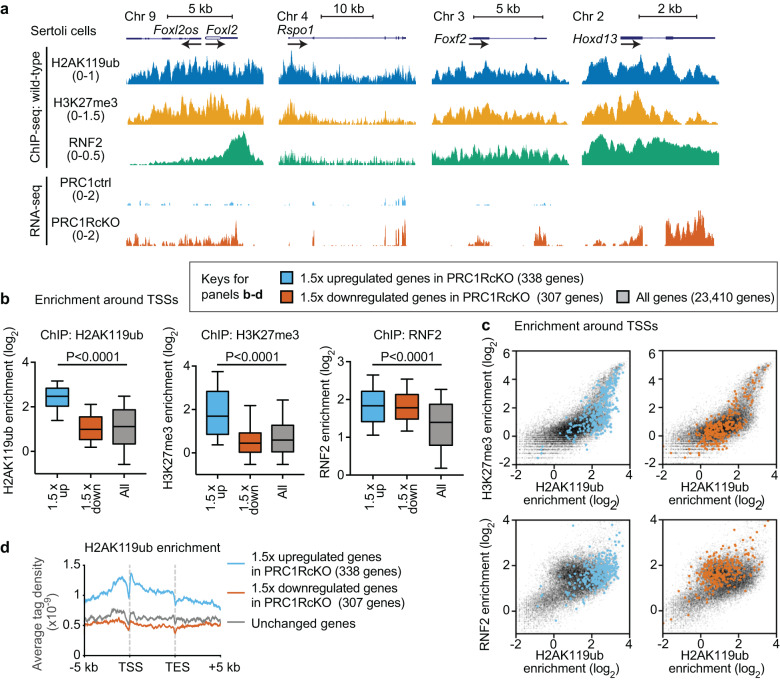
In Sertoli cells, Polycomb-mediated marks are enriched on genes required for granulosa cells. **a** Genome track views of representative genes in the female gene regulatory network. ChIP-seq enrichment in wild-type Sertoli cells is shown (top); RNA-seq peaks in PRC1ctrl and PRC1RcKO Sertoli cells are shown (bottom). Arrows indicate the locations and directions of TSSs. The numbers in brackets show the data ranges of normalized enrichment. **b** Box-and-whisker plots showing distributions of enrichment for ChIP-seq data. Central bars represent medians, the boxes encompass 50% of the data points, and the whiskers indicate 90% of the data points. *P* values, Mann-Whitney U tests. **c** Scatter plots showing ChIP-seq enrichment (±2 kb around TSSs) of indicated modifications on genes upregulated (left panels) and downregulated (right panels) in Sertoli cells. The distribution of all genes is shown with gray dots. **d** Average tag densities of H2AK119ub ChIP-seq enrichment. Color keys for **b-d** are shown in the panel.

Track views of these ChIP-seq data showed that H2AK119ub, H3K27me3, and RNF2 were enriched at *Foxl2* and *Rspo1* loci (Fig. 4a). Furthermore, enrichment of these three marks was found in other loci such as *Foxf2*, the mutation of which appears in patients with disorders of sex development [47], and *Hoxd13*, which is involved in female reproductive tract development [48]. These results suggest that PRC1 works with PRC2 to suppress female genes as well as developmental regulators such as *Fox* and *Hox* genes, which are classic targets of Polycomb-mediated gene repression [49]. As for male-specific gene loci, *Sox9*, *Dmrt1*, and *Dhh* were downregulated upon loss of PRC1, H3K27me3 was largely depleted, and H2AK119ub was present on portions of these loci (Supplementary Fig. 9a). These results suggest that PRC1 does not work with PRC2 on male gene loci, consistent with the active state of these loci in wild-type Sertoli cells.

Further, we found that RNF2 was enriched at the *Cdkn2a*/*Ink4a*/*Arf* locus (Supplementary Fig. 9b), which encodes cell cycle inhibitors and is a classic target locus of Polycomb proteins [50]. H2AK119ub and H3K27me3 were present at the *Cdkn2a* locus, and *Cdkn2a* was derepressed upon the loss of PRC1 (Supplementary Fig. 9b). Taken together, our data demonstrate that *Cdkn2a* is a direct PRC1 target in Sertoli cells and suggest that the change in proliferation in PRC1-depleted Sertoli cells is the direct result of PRC1 loss.

To determine the features of genome-wide gene repression mediated by Polycomb complexes, we analyzed the enrichment of H2AK119ub, H3K27me3, and RNF2 on the upregulated genes in PRC1RcKO Sertoli cells. H2AK119ub, H3K27me3, and RNF2 were all significantly enriched on the TSSs of upregulated genes in PRC1RcKO Sertoli cells as compared to all genes in the genome (Fig. 4b, Supplementary File 2). Additional enrichment analysis confirmed the co-enrichment of H2AK119ub and H3K27me3 (Fig. 4c, upper panels) as well as co-enrichment of H2AK119ub and RNF2 (Fig. 4c, lower panels) on upregulated genes in PRC1RcKO Sertoli cells. Furthermore, average tag density analysis confirmed that enrichment of H2AK119ub on upregulated genes in PRC1RcKO Sertoli cells occurred in upstream regions, gene bodies, and downstream regions with the highest enrichment near TSSs (Fig. 4d). We found a similar distribution of H3K27me3 around the gene bodies of upregulated genes in PRC1RcKO Sertoli cells (Supplementary Fig. 9c). Together, these results confirmed the genome-wide, global functions of PRC1 in direct regulation of gene repression in Sertoli cells.

### PRC1 globally inactivates the female gene regulatory network in postnatal Sertoli cells

**Fig. 5:**
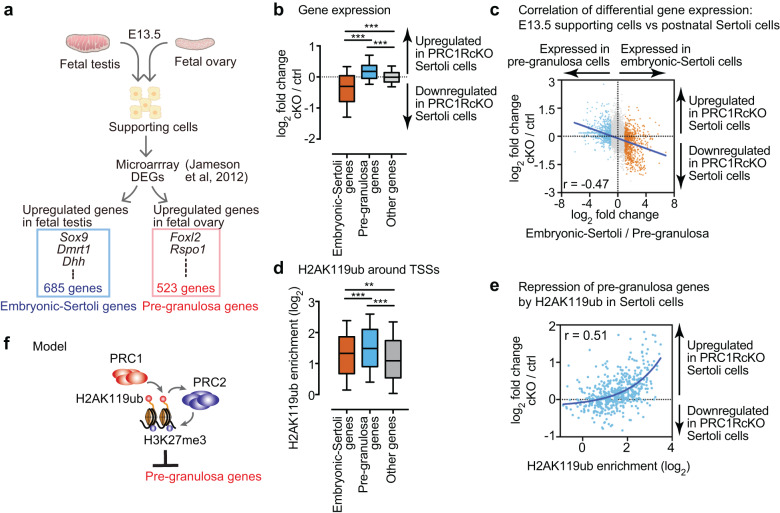
PRC1 inactivates the female gene regulatory network in Sertoli cells. **a** Schematic showing method for identification of pre-granulosa genes and embryonic-Sertoli genes based on a previous microarray analysis [51]. **b** Box-and-whisker plots showing distributions of RNA-seq data of PRC1RcKO Sertoli cells. Central bars represent medians, the boxes encompass 50% of the data points, and the whiskers indicate 90% of the data points. ***P < 0.0001, Mann-Whitney U tests. **c** Scatter plots showing the correlation between RNA-seq data for genes regulated in E13.5 supporting cells and P7 Sertoli cells. A Pearson correlation value (r) is shown in the panel. A linear trendline is shown in blue. **d** Box-and-whisker plots showing distributions of enrichment for H2AK119ub ChIP-seq data in wild-type Sertoli cells. Central bars represent medians, the boxes encompass 50% of the data points, and the whiskers indicate 90% of the data points. ***P < 0.0001, **P < 0.005, Mann-Whitney U tests. **e** Scatter plots showing the correlation between ChIP-seq enrichment (±2 kb around TSSs) for H2AK119ub ChIP-seq data in wild-type Sertoli cells and expression changes of pre-granulosa genes in PRC1RcKO Sertoli cells. A Pearson correlation value (r) is shown in the panel. A linear trendline is shown in blue. **f** Model of PRC1-dependent regulation of PRC2 in suppression of pre-granulosa genes.

Previous studies have suggested that sex determination is canalized by the interconnected, antagonistic network of genes both in males and in females that are controlled by feedback mechanisms [3]. Since Polycomb is implicated in the maintenance of sex-specific gene regulatory networks through the PRC2-mediated mark H3K27me3 [19], we hypothesized that PRC1 globally inactivates the female gene regulatory network in postnatal Sertoli cells. Because male- and female-specific gene networks are regulated immediately after sex determination during fetal stages [51], we reasoned that female gene network suppression in fetal Sertoli cells is maintained by PRC1 in postnatal Sertoli cells. To test this hypothesis, we identified 523 specifically expressed genes in E13.5 granulosa cells [51], termed “pre-granulosa genes” (Fig. 5a, Supplementary Fig. 10a, Supplementary File 3), and examined their expression in postnatal Sertoli cells. We found that pre-granulosa genes were upregulated in PRC1RcKO postnatal Sertoli cells as compared to other genes (Fig. 5b). On the other hand, 685 specifically expressed genes in E13.5 Sertoli cells [51], termed “embryonic-Sertoli genes” (Fig. 5a, Supplementary Fig. 10a, Supplementary File 3), were downregulated in PRC1RcKO postnatal Sertoli cells as compared to other genes (Fig. 5b). We further examined the correlation of gene expression of each individual gene genome-wide and found that pre-granulosa genes were highly correlated with upregulated genes in PRC1RcKO postnatal Sertoli cells, while embryonic-Sertoli genes were highly correlated with downregulated genes in PRC1RcKO postnatal Sertoli cells (Fig. 5c). These results suggest that PRC1 maintains suppression of the female gene regulatory network, which is initiated at the time of sex determination and is maintained throughout the development of postnatal Sertoli cells.

We next sought to determine whether PRC1 directly suppresses the female gene regulatory network in postnatal Sertoli cells. H2AK119ub was significantly enriched on TSSs of pre-granulosa genes compared to other genes and embryonic-Sertoli genes in wild-type Sertoli cells (Fig. 5d). Among pre-granulosa genes, enrichment of H2AK119ub in wild-type Sertoli cells was positively correlated with upregulated genes in PRC1RcKO postnatal Sertoli cells (Fig. 5e). We further identified the enrichment of H3K27me3 on TSSs of pre-granulosa genes in wild-type postnatal Sertoli cells (Supplementary Fig. 10b). Among pre-granulosa genes, enrichment of H3K27me3 in wild-type Sertoli cells was positively correlated with upregulated genes in PRC1RcKO postnatal Sertoli cells (Supplementary Fig. 10c). Together, these data suggest that PRC1 globally inactivates the female gene regulatory network in collaboration with PRC2 in postnatal Sertoli cells (Fig. 5f).

**Fig. 6:**
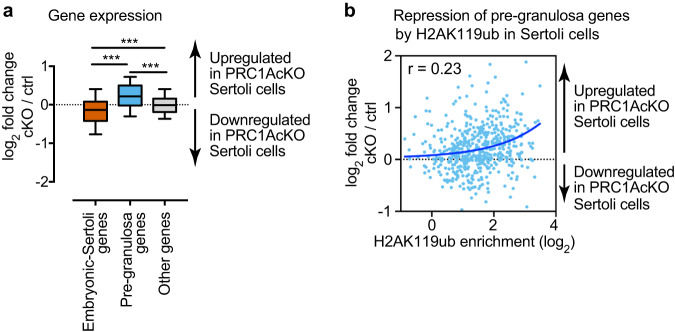
PRC1-dependent regulation of the female gene regulatory network in PRC1AcKO Sertoli cells. **a** Box-and-whisker plots showing distributions of RNA-seq data of PRC1AcKO Sertoli cells. Central bars represent medians, the boxes encompass 50% of the data points, and the whiskers indicate 90% of the data points. *** *P* < 0.0001, Mann-Whitney U tests. **b** Scatter plots showing the correlation between ChIP-seq enrichment (±2 kb around TSSs) for H2AK119ub ChIP-seq data in wild-type Sertoli cells and expression changes of pre-granulosa genes in PRC1AcKO Sertoli cells. A Pearson correlation value (r) is shown in the panel. A linear trendline is shown in blue.

To independently confirm this conclusion, we also performed an RNA-seq analysis of Sertoli cells isolated from P7 PRC1AcKO testes and littermate controls. This experiment enabled us to assess the gene expression profiles of Sertoli cells without any adverse effects of culture after isolation from testes. We performed RNA-seq for two independent biological replicates and confirmed reproducibility between biological replicates (Supplementary Fig. 11a, b, Supplementary File 4). Because approximately 60% of PRC1AcKO Sertoli cells at P7 were depleted of RNF2 (i.e., ~40% of them escaped and replenished Sertoli cells from Cre*-*mediated recombination: Supplementary Fig. 2b), we anticipated that there could be a mild phenotype in gene expression in PRC1AcKO Sertoli cells at P7. As we expected, pre-granulosa genes were derepressed in PRC1AcKO Sertoli cells at P7 (Fig. 6a). Among pre-granulosa genes, there is a modest correlation between enrichment of H2AK119ub in wild-type Sertoli cells and upregulated genes in PRC1AcKO Sertoli cells at P7 (Fig. 6b). Consistent with this observation, a principal component analysis revealed that the direction of changes in PRC1 conditional deletion is consistent between PRC1AcKO and PRC1RcKO Sertoli cells (Supplementary Fig. 11c). Although there appears to be a difference in gene expression between isolated Sertoli cells without culture (PRC1AcKO Sertoli cells) and with culture (PRC1RcKO Sertoli cells), these results suggest that the loss of PRC1 universally caused derepression of the female gene regulatory network regardless of the effect of culture conditions. All in all, we conclude that PRC1 globally inactivates the female gene regulatory network in Sertoli cells.

## Discussion

**Fig. 7:**
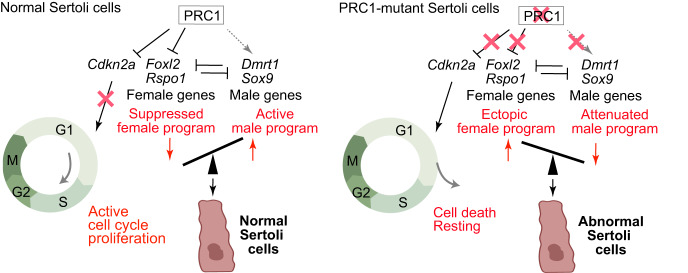
Model of PRC1’s function in Sertoli cells. PRC1 has two major molecular functions in Sertoli cells: control of proliferation and suppression of the female gene regulatory network.

In this study, we demonstrated that PRC1 is required for proliferation of Sertoli cells and suppression of the non-lineage-specific gene expression program and the female gene regulatory network. Among these functions, we propose that suppression of the female gene regulatory network is the key mechanism to ensure male fate in addition to canonical functions of PRC1, which controls proliferation and suppression of non-lineage-specific gene expression programs. Although we did not observe complete infertility using *Amh*-Cre (presumably due to the repopulation of Sertoli cells that escaped Cre-mediated recombination), smaller testis size and abnormal tubule organization of cKO testes (Fig. 1) suggest that PRC1 is critical for physiological functions of Sertoli cells. Below, we discuss two molecular aspects underlying these physiological phenotypes of PRC1-depleted Sertoli cells: control of proliferation and suppression of the female gene regulatory network (Fig. 7).

The proliferation of Sertoli cells is a critical determinant of testicular functions because the number of Sertoli cells dictates testicular output, in particular spermatogenesis [42]. Rapid proliferation is a prominent feature of juvenile Sertoli cells. In general, Polycomb proteins are associated with the cell cycle checkpoint by directly suppressing the tumor suppressor locus *Cdkn2a*/*Ink4a*/*Arf* [50], which functions as a barrier to cancer transformation [52]. Therefore, enhanced Polycomb activity is a frequent feature of human tumors. We found that *Cdkn2a* was derepressed in PRC1RcKO Sertoli cells (approximately 2.5-fold upregulation: PRKM value for PRC1RcKO: 32.1 v.s. PRC1ctrl: 13.0) and in PRC1AcKO Sertoli cells (approximately 2.6-fold upregulation: PRKM value for PRC1RcKO: 24.6 v.s. PRC1ctrl: 9.6). Therefore, our results suggest that PRC1 directly promotes the rapid proliferation of Sertoli cells by suppressing *Cdkn2a*. The function of PRC1 in Sertoli cell proliferation is distinct from Polycomb functions in testicular germ cells, where PRC1 depletion does not alter the proliferation of germ cells [34]. This may be due to the fact that PRC1 and PRC2 are not required for suppression of *Cdkn2a* in germ cells [34, 53]. The functional difference in testicular germ cells and Sertoli cells highlights the context-dependent functions of Polycomb proteins.

Another critical function of PRC1 in Sertoli cells is the suppression of the female gene regulatory network. DMRT1 is a critical transcription factor that suppresses the expression of female genes [8]. While more than 10-fold upregulation of female genes (e.g., *Rspo1* and *Foxl2*) was observed for *Dmrt1* mutants [8], the degree of upregulation of these genes was modest in PRC1RcKO Sertoli cells (Fig. 3). This finding, combined with genetic evidence, indicates that DMRT1 could be the direct regulator of suppression of female genes, while PRC1-mediated mechanisms could be a maintenance mechanism in response to the primary silencing mechanisms determined by DMRT1. Consistent with this notion, we did not observe complete sex reversal in PRC1RcKO testes (Supplementary Fig. 7). Another possibility why we saw a mild phenotype compared to that of *Dmrt1* mutants is compensation by PRC1-independent suppression mechanisms. While a portion of H3K27me3 is regulated by variant PRC1 [27, 28], another portion of H3K27me3 is mediated by PRC2 that is not downstream of PRC1 [54]. These PRC2-mediated mechanisms or other silencing machinery may be responsible for the suppression of female genes. Of note, in our ChIP-seq experiments, H2AK119ub and H3K27me3 are specifically enriched on upregulated genes in PRC1RcKO Sertoli cells (Fig. 4b), while RNF2 is abundant on both down and upregulated genes. This data suggests that PRC1 and PRC2 are coordinated on these target genes, raising the possibility that variant PRC1 complexes, which direct PRC2 activities, may serve as major regulators of the female pathway. Specifically, our *Amh*-*Cre* model clarified the function of PRC1 in the maintenance of the Sertoli cell-specific gene expression program after E14.5. Nevertheless, due to the recovery of Sertoli cells that escaped PRC1 depletion in our *Amh*-*Cre* model, additional experiments are required to test the model that PRC1, in concert with PRC2, is required for Sertoli maintenance in adult testes.

The notion that Polycomb regulates the female gene regulatory network has been supported by other recent evidence. In bipotential precursor cells, genes involved in sex determination are marked with bivalent chromatin domains [19] that are prevalent in pluripotent stem cells and in germ cells [55–60]. The maintenance of the male fate was explained by the persistence of H3K27me3 on silent female genes in Sertoli cells [19]. Consistent with PRC1’s function found in the current study, Polycomb-mediated silencing may globally suppress the female gene regulatory network. We found that H2AK119ub was largely associated with this group of female genes (Fig. 5). Therefore, it would be interesting to speculate that antagonistic male and female networks can be directly coordinated by Polycomb protein functions, including the strong feedback mechanism underlying both networks. Of note, upregulated genes in PRC1RcKO Sertoli cells were highly enriched with both H2AK119ub and H3K27me3, while enrichment of RNF2 is comparable between both groups of upregulated and downregulated genes in PRC1RcKO Sertoli cells (Fig. 4b). Therefore, it is possible that activities of both PRC1 and PRC2 are coordinated to suppress the female gene regulatory network. These possibilities raise several outstanding questions to be addressed in future studies. What are the functions of another Polycomb complex, PRC2, and of each Polycomb complex component, including CBX2, in postnatal Sertoli cells? To test if PRC2 activity is regulated downstream of PRC1, it would be important to examine the activity of PRC2 in PRC1-depleted Sertoli cells. Other key questions include: what is the function of Polycomb in female granulosa cells, especially in the suppression of the male gene regulatory network? Does Polycomb underlie the feedback regulation of each network to define sexual identity? Our current study provides a foundation to explore these questions.

## Methods

### Animals

Generation of mutant *Ring1* and *Rnf2* floxed alleles was previously reported [61]. *Amh-*Cre transgenic mice were purchased from The Jackson Laboratory (Stock No: 007915) [32]. A conditional PRC1 deletion mouse model was generated using *Amh*-*Cre* in a background of *Ring1*-knockout (KO) mice (*Amh*-Cre; *Rnf2*cKO; *Ring1*-KO: termed PRC1^*Amh*-Cre^cKO: PRC1AcKO). Littermates that harbored floxed alleles for *Rnf2* on a *Ring1*-KO background without *Amh*-Cre (*Rnf2*^floxed/floxed^; *Ring1*-KO) were used as controls (termed PRC1 control: PRC1ctrl).

ROSA26-CreERT2 mice were purchased from The Jackson Laboratory (Stock No: 008463) [62]. A tamoxifen-inducible PRC1-depletion mouse model was generated using the ROSA26-CreERT2 allele (ROSA26-CreERT2; *Rnf2*^floxed/floxed^; *Ring1*-KO: termed PRC1 ^ROSA26-CreERT2^: PRC1RcKO). Littermates that harbored floxed alleles for *Rnf2* on a *Ring1*-KO background without ROSA26-CreERT2 (*Rnf2*^floxed/floxed^; *Ring1*-KO) were used as controls (termed PRC1 control: PRC1ctrl).

A minimum of three independent mice were analyzed for each experiment. The Institutional Animal Care and Use Committee (IACUC) at Cincinnati Children’s Hospital Medical Center approved this work: protocol no. IACUC2018-0040. Fertility tests were performed with 6-week-old CD1 female mice (purchased from Charles River). At least 2 female mice were bred with a male mouse for 2 weeks, and fertility was evaluated by the ratio of pregnant to total female mice and the number of pups.

### Hormone tests

Blood samples were collected from C57BL/6 N mice aged 8-11 weeks. Serum was separated immediately and stored at -20 °C. Hormone assays, including testosterone, estradiol, and follicle-stimulating hormone, were performed by the Center for Research in Reproduction at the University of Virginia.

### Sertoli cell isolation

Sertoli cells were isolated as previously described with minor modifications [63] and collected from C57BL/6 N mice aged 6–8 days. Testes were collected in a 24-well plate in Dulbecco’s Modified Eagle Medium (DMEM) supplemented with GlutaMax (Thermo Fisher Scientific), non-essential amino acids (NEAA) (Thermo Fisher Scientific), and penicillin and streptomycin (Thermo Fisher Scientific). After removing the *tunica albuginea* membrane, testes were digested with collagenase (1 mg/ml) at 34 °C for 20 min to remove interstitial cells, then centrifuged at 188×*g* for 5 min. Tubules were washed with medium and then digested with trypsin (2.5 mg/ml) at 34 °C for 20 min to obtain a single-cell suspension. To remove KIT^+^ spermatogonia, cells were washed with magnetic cell-sorting (MACS) buffer (PBS supplemented with 0.5% BSA and 5 mM EDTA) and incubated with CD117 (KIT) MicroBeads (Miltenyi Biotec) on ice for 20 min. Cells were separated by an autoMACS Pro Separator (Miltenyi Biotec) with the program “possel.” Cells in the flow-through fraction were washed with MACS buffer and incubated with CD90.2 (THY1) MicroBeads (Miltenyi Biotec) on ice for 20 min to remove THY1^+^ spermatogonia. Cells were separated by an autoMACS Pro Separator (Miltenyi Biotec) with the program “posseld.” Cells in the flow-through fraction were washed and plated in a 6-well plate for 1 h in medium supplemented with 10% fetal bovine serum, which promotes adhesion of Sertoli cells. Purity was confirmed by immunostaining.

For PRC1RcKO, cells were cultured for 4 days with 1 µM 4-OHT dissolved in ethanol or ethanol only as vehicle control in Dulbecco’s Modified Eagle Medium (DMEM) supplemented with GlutaMax (Thermo Fisher Scientific), non-essential amino acids (NEAA) (Thermo Fisher Scientific), and penicillin and streptomycin (Thermo Fisher Scientific). The same medium was replaced 2 days after the initiation of the culture.

### Histological analysis and immunofluorescence

For the preparation of testicular paraffin blocks, testes were fixed with 4% paraformaldehyde (PFA) overnight at 4 °C with gentle inverting. For EdU-incorporation, 100 μl of 20 mM EdU in PBS per 10 g of mouse body weight was intraperitoneally injected using a sterile 32 G needle attached to a 1-ml syringe 24 h prior to the harvesting of testes. Testes were dehydrated and embedded in paraffin. For immunofluorescence analysis of testicular sections, antigen retrieval was performed by boiling the slides in target retrieval solution (DAKO) for 10 min and letting the solution cool for 30 min. Sections were blocked with Blocking One Histo (Nacalai) for 1 h at room temperature and then incubated with primary antibodies overnight at 4 °C. The list of primary antibodies used for immunostaining is described in Supplementary File 5. The resulting signals were detected by incubation with secondary antibodies conjugated to Alexa Fluor dyes (Thermo Fisher Scientific or Jackson ImmunoResearch). For detection of apoptotic cells, terminal deoxynucleotidyl transferase dUTP nick end labeling (TUNEL) was performed using the In Situ Cell Death Detection Kit (Roche) according to the manufacturer’s instructions. Sections were counterstained with DAPI. Images were obtained via a laser scanning confocal microscope A1R (Nikon) and processed with NIS-Elements (Nikon) and ImageJ (National Institutes of Health) [64].

### Western blotting

For western blotting, total proteins from Sertoli cells were dissolved in SDS-PAGE sample buffer (62.5 mM Tris-HCl pH 6.8, 2% SDS, 10% glycerol, 5% β-mercaptoethanol, and 0.02% bromophenol blue) with Benzonase (NEB), separated by SDS-PAGE, and blotted to an Immobilon-P PVDF Membrane (Millipore) by Trans-Blot^®^ Turbo™ Transfer System (BioRad). The membranes were blocked in StartingBlock™ T20 (TBS) Blocking Buffer (Thermo Fisher Scientific) for 30 min, probed with a primary antibody in 1/20 diluted blocking buffer at 4^o^C overnight, and then incubated with an HRP-conjugated secondary antibody for 1 h at room temperature. The list of primary antibodies used for western blotting is described in Supplementary File 5. Blots were developed using Immobilon Western Chemiluminescent HRP Substrate (Millipore) and exposed onto Super RX-N X-ray film (Fujifilm). To reprobe blots, membranes were incubated in Restore™ Western Blot Stripping Buffer (Thermo Fisher Scientific).

### RNA-seq library preparation

Total RNA was purified from 1 × 10^6^ Sertoli cells using an RNeasy Micro Kit (Qiagen) according to the manual provided. RNA quality and quantity were checked using Bioanalyzer (Agilent) and Qubit (Life Technologies), respectively. To amplify RNA samples and create double-stranded cDNA, NuGEN Ovation RNA-seq System Version 2 was used. Libraries were then created with the Nextera XT DNA Library Preparation Kit (Illumina).

### Crosslinking ChIP-seq library preparation with the ChIPmentation method and sequencing

We used ~2–4 × 10^6^ Sertoli cells isolated from wild-type testes. Cells were suspended in chilled 1× PBS. One-eleventh volume of crosslinking solution (50 mM HEPES-NaOH pH 7.9, 100 mM NaCl, 1 mM EDTA, 0.5 mM EGTA, and 8.8% formaldehyde) was added to the cell suspension and incubated on ice for 8 min. One-twentieth volume of 2 M glycine was added to the cell suspension and incubated at room temperature for 5 min to stop the reaction. Cells were washed twice with PBS, frozen at -80 °C, and lysed at 4 °C for 10 min each in ChIP lysis buffer 1 (50 mM HEPES pH 7.9, 140 mM NaCl, 10% glycerol, 0.5% IGEPAL-630, 0.25% Triton X-100). After centrifugation at 2000× *g* for 10 min at 4 °C, pellets were resuspended with ChIP lysis buffer 2 (10 mM Tris-HCl pH 8.0, 200 mM NaCl, 1 mM EDTA, 0.5 mM EGTA) and incubated at 4 °C for 10 min. After centrifugation at 2000× *g* for 10 min at 4 °C, pellets were washed with TE containing 0.1% SDS and protease inhibitors (Sigma; 11836145001) and resuspended with the same buffer. Chromatin was sheared to approximately 200–500 bp by sonication using a Covaris sonicator at 10% duty cycle, 105 pulse intensity, and 200 bursts for 2 min. Sheared chromatin was cleared by centrifugation at 20,000× *g* for 20 min, followed by pre-incubation with Dynabeads Protein G (Thermo Fisher Scientific). Chromatin immunoprecipitation was carried out on an SX-8X IP-STAR compact automated system (Diagenode). Briefly, Dynabeads Protein G was pre-incubated with 0.1% BSA for 2 h. Then, the cleared chromatin was incubated with beads conjugated to antibodies against H2AK119ub, H3K27me3, or RNF2 at 4 °C for 8 h (a list of primary antibodies used for ChIP-seq is included in Supplementary File 5), washed sequentially with wash buffer 1 (50 mM Tris-HCl pH 8.0, 150 mM NaCl, 1 mM EDTA, 0.1% SDS, 0.1% NaDOC, and 1% Triton X-100), wash buffer 2 (50 mM Tris-HCl pH 8.0, 250 mM NaCl, 1 mM EDTA, 0.1% SDS, 0.1% NaDOC, and 1% Triton X-100), wash buffer 3 (10 mM Tris-HCl pH 8.0, 250 mM LiCl, 1 mM EDTA, 0.5% NaDOC, and 0.5% NP-40), wash buffer 4 (10 mM Tris-HCl pH 8.0, 1 mM EDTA, and 0.2% Triton X-100), and wash buffer 5 (10 mM Tris-HCl).

DNA libraries were prepared through the ChIPmentation method [65]. Briefly, beads were resuspended in 30 μl of Tagmentation Buffer (10 mM Tris-HCl pH 8.0 and 5 mM MgCl_2_) containing 1 μl Tagment DNA Enzyme from the Nextera DNA Sample Prep Kit (Illumina) and incubated at 37 °C for 10 min in a thermal cycler. The beads were washed twice with 150 μl cold wash buffer 1, incubated with elution buffer (10 mM Tris-HCl pH 8.0, 1 mM EDTA, 250 mM NaCl, 0.3% SDS, 0.1 μg/μl Proteinase K) at 42 °C for 30 min, and then incubated at 65 °C for another 5 h to reverse crosslinking. DNA was purified with the MinElute Reaction Cleanup Kit (Qiagen) and amplified with NEBNext Ultra II Q5 Master Mix (NEB). Amplified DNA was purified by Agencourt AMPure XP (Beckman Coulter). Afterward, DNA fragments in the 250- to 500-bp size range were prepared by agarose gel size selection. DNA libraries were adjusted to 5 nM in 10 mM Tris-HCl pH 8.0 and sequenced with an Illumina HiSeq 2500.

### ChIP-sequencing, RNA-sequencing, and data analysis

Data analysis for both ChIP-seq and RNA-seq was performed in the BioWardrobe Experiment Management System (https://github.com/Barski-lab/biowardrobe [66]). For RNA-seq analysis, reads were aligned by STAR (version STAR_2.5.3a) [67] with default arguments except --outFilterMultimapNmax 1 and --outFilterMismatchNmax 2. The --outFilterMultimapNmax parameter was used to allow unique alignments only, and the --outFilterMismatchNmax parameter was used to allow a maximum of 2 errors. NCBI RefSeq annotation from the mm10 UCSC genome browser 76 was used, and canonical TSSs (1 TSS per gene) were analyzed. All reads from the resulting .bam files were split for related isoforms with respect to RefSeq annotation. Then, the EM algorithm was used to estimate the number of reads for each isoform. To detect differentially expressed genes between two biological samples, a read count output file was input to the DESeq2 package (version 1.16.1); then, the program functions DESeqDataSetFromMatrix and DESeq were used to compare each gene’s expression level between two biological samples. Differentially expressed genes were identified through binominal tests, thresholding Benjamini-Hochberg- adjusted *P* values to <0.01. To perform gene ontology analyses, the functional annotation clustering tool in DAVID (version 6.8) was used, and a background of all mouse genes was applied. Biological Process term groups with a significance of *P* < 0.05 (modified Fisher’s exact test) were considered significant. To analyze the relationship among all RNA-seq data in this study, the similarity among them was visualized by a PCA plot using SeqMonk (Babraham Institute).

For ChIP-seq analysis, reads were aligned to the mouse genome (mm10) with Bowtie (version 1.0.0 [68]), assigned to RefSeq genes (which have one annotation per gene) using the BioWardrobe algorithm, and displayed on a local mirror of the UCSC genome browser as coverage. Peaks of H2AK119ub, H3K27me3 and RNF2 enrichment were identified using MACS2 (version 2.0.10.20130712 [69]). Pearson correlations for the genome-wide enrichment of the peaks among ChIP-seq library replicates were analyzed using SeqMonk (Babraham Institute). Average tag density profiles were calculated around gene bodies, including 5 kb upstream and 5 kb downstream of the genes. The resulting graphs were smoothed in 200-bp windows. Enrichment levels for ChIP-seq experiments were calculated for 4-kb windows, promoter regions of genes (±2 kb surrounding TSSs), and enhancer regions. To normalize tag values, read counts were multiplied by 1,000,000 and then divided by the total number of reads in each nucleotide position. The total amount of tag values in promoter or enhancer regions was calculated as enrichment.

Microarray data were analyzed using the processed data [51]. Differentially expressed genes were identified through a p-value cutoff of 0.05 and a fold change cutoff of 2 for the comparison between E13.5 XX supporting cells and E13.5 XY supporting cells. Highly expressed genes in E13.5 XY supporting cells and in E13.5 XX supporting cells were termed as "embryonic-Sertoli genes" and "pre-granulosa genes," respectively.

## Supplementary information

Supplementary File 1

Supplementary File 2

Supplementary File 3

Supplementary File 4

Supplementary File 5

SupplementaryFigures

Reproducibility Checklist

SupplementaryMaterial

## Data Availability

RNA-seq data and ChIP-seq data reported in this study were deposited to the Gene Expression Omnibus (accession no. GSE167516: a token to access the private data for peer review is cbyrquqcdpkthcl).
