# Supplementary material for: PRC1 suppresses a female gene regulatory network to ensure testicular differentiation
Source: Cell Death Dis. 2023 Aug 4;14(8):501. doi: 10.1038/s41419-023-05996-6 (PMC10403552; doi:10.1038/s41419-023-05996-6)
Supplement: Supplementary file 6 — SupplementaryFigures [file 41419_2023_5996_MOESM6_ESM.pdf]

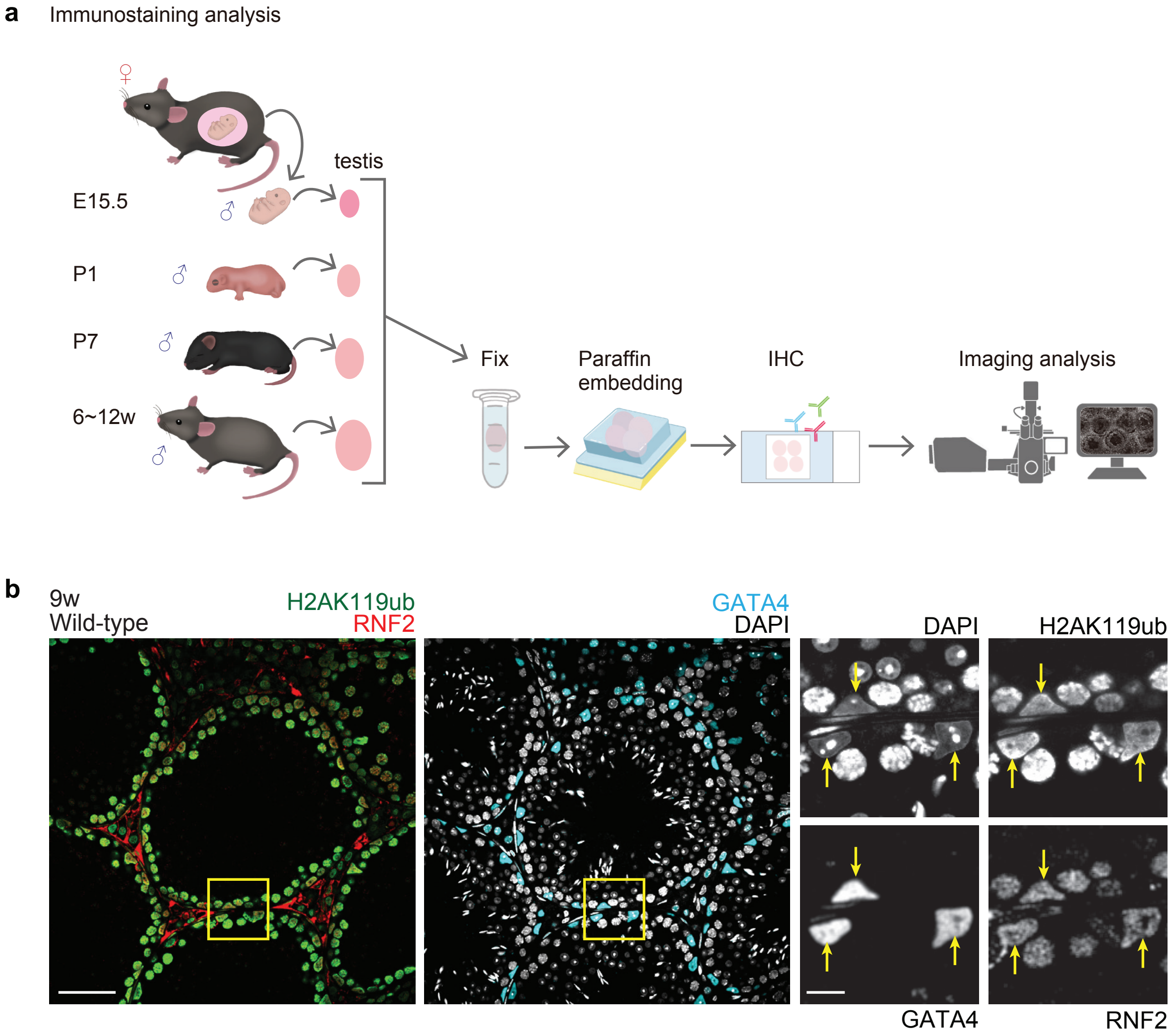

Supplementary Fig. 1. Localization of H2AK119ub and RNF2 in wild-type testes at 9 weeks of age.
(**a**) Schematic of the experimental steps of immunostaining analysis.
(**b**) Localization of H2AK119ub, GATA4, and RNF2 in wild-type testis at 9 weeks of age (9w). A region marked by yellow squares is magnified in the right panel. GATA4^{+} Sertoli cells are shown with yellow arrows. 50 µm. Bars in the magnified panels: 10 µm.

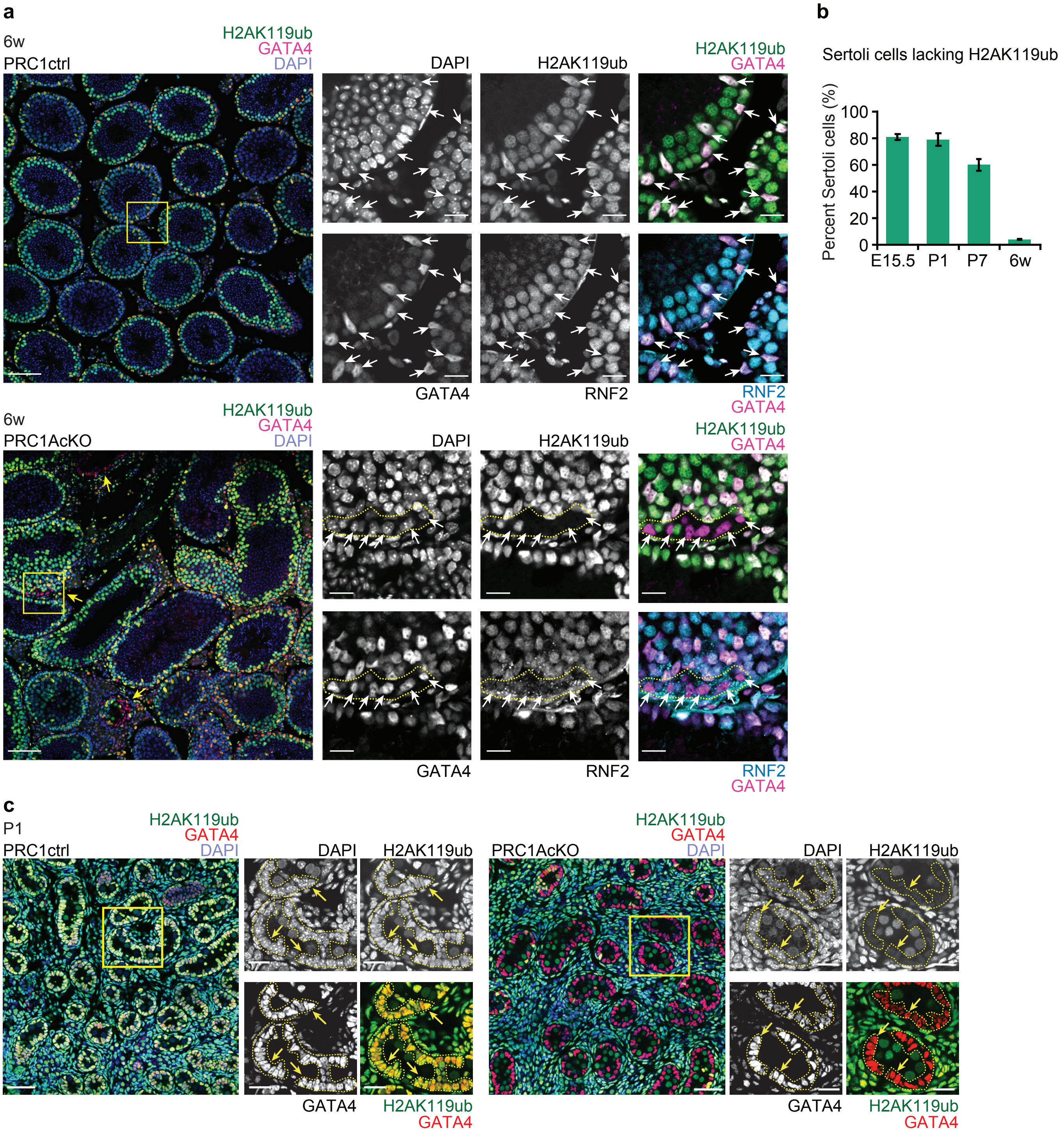

Supplementary Fig. 2. Deletion efficiency of *Amh*-Cre driven deletion of RNF2 in Sertoli cells and testicular degeneration of mutant testes at 6 weeks of age.

(**a**) Localization of H2AK119ub, GATA4, and RNF2 in PRC1ctrl and PRC1AcKO at 6 weeks of age (6w). GATA4^{+} Sertoli cells in mutants are shown with white arrows.

(**b**) The efficiency of *Amh*-Cre mediated RNF2 depletion in Sertoli cells at E15.5, P1, P7, and 6w. Data are presented as mean values ± SEM. Four independent mice were examined.

(**c**). Localization of H2AK119ub and GATA4 in PRC1ctrl and PRC1AcKO at embryonic day P1. Sertoli cells are shown with yellow arrows. Regions marked by yellow squares are magnified in the right panels. Bars in the large panels: 50 µm. Bars in the magnified panels: 20 µm.

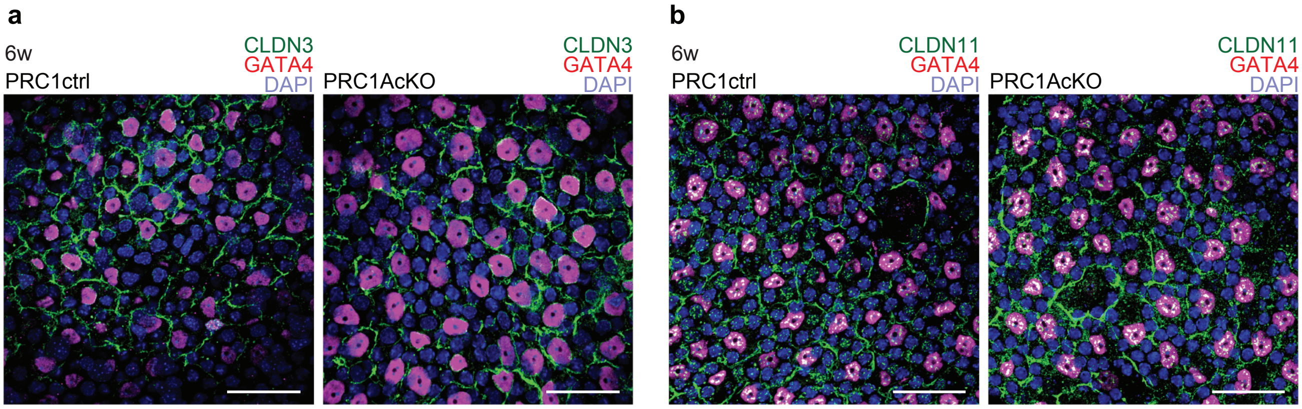

Supplementary Fig. 3. The distribution of tight junction proteins CLDN3 and CLDN11 did not change in 6-week-old PRC1AcKO testes.
(**a, b**) Localization of CLDN3 (**a**) and CLDN11 (**b**) with GATA4 in PRC1ctrl and PRC1AcKO testes at 6 weeks of age (6w). Bars: 50 µm.

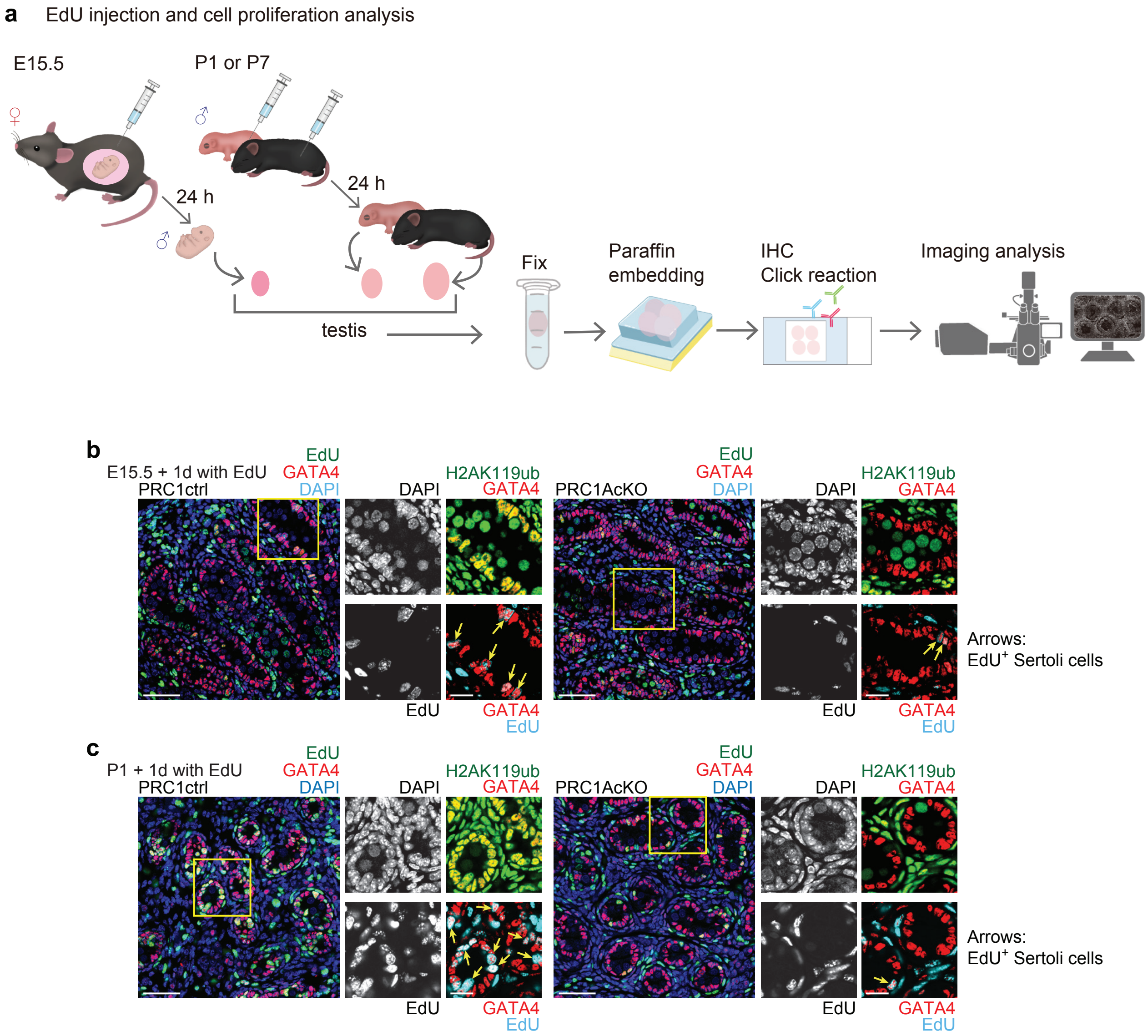

Supplementary Fig. 4. EdU incorporation in PRC1ctrl and PRC1AcKO Sertoli cells.

(**a**) Schematic of the experimental steps of EdU injection and cell proliferation analysis.

(**b, c**) Testicular sections of the indicated genotypes 1 day following the injection of EdU into males at E15.5 (**b**) and P1 (**c**). The presence of EdU^{+} Sertoli cells (yellow arrows) was decreased in PRC1AcKO testes. Regions marked by yellow squares are magnified in the right panels. Bars in the large panels: 50 µm. Bars in the magnified panels: 20 µm

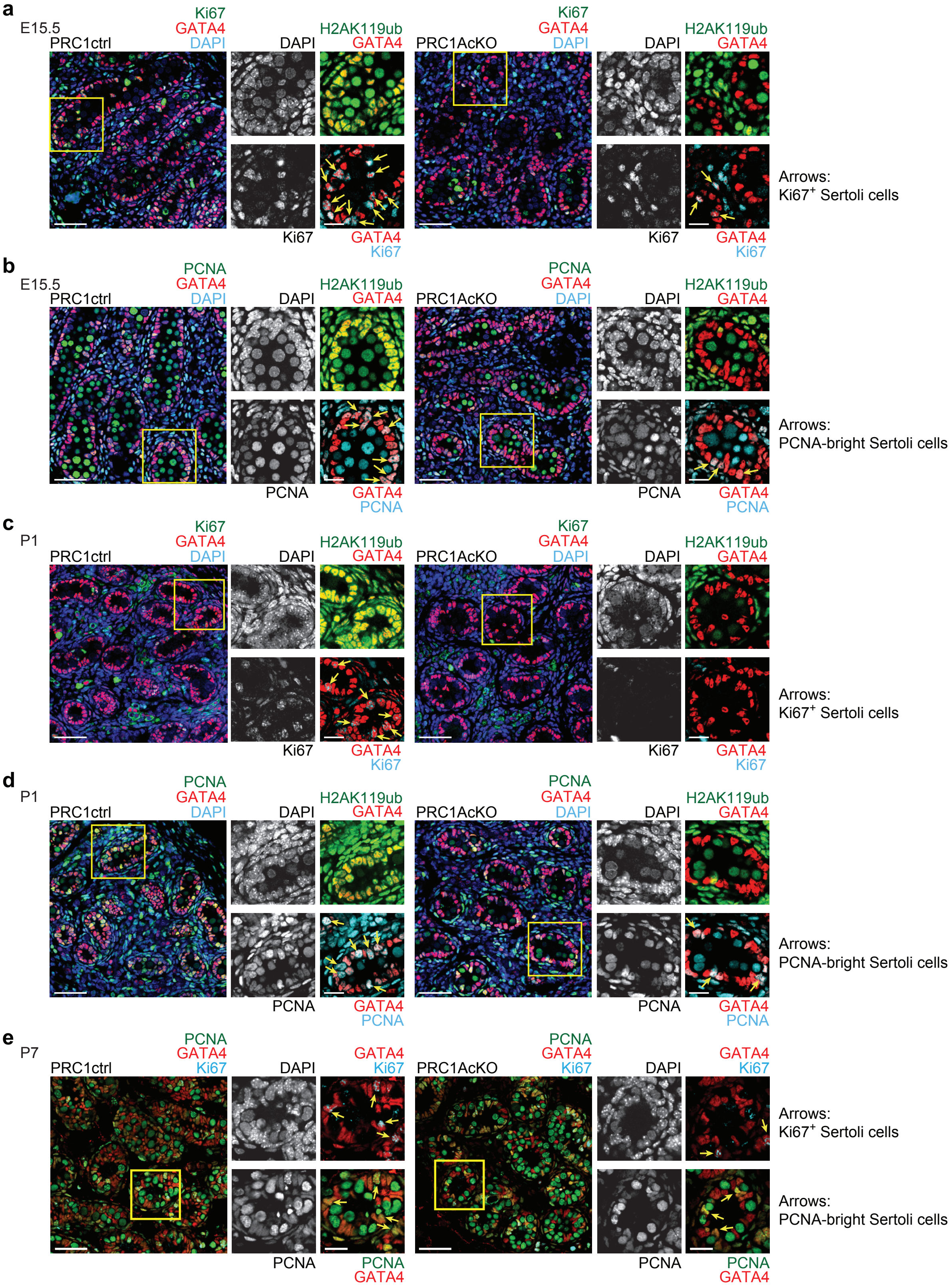

Supplementary Fig. 5. Ki67 and PCNA localization in PRC1ctrl and PRC1AcKO Sertoli cells.
(**a-e**) Distribution of Ki67 or PCNA on GATA4^{+} Sertoli cells in testicular sections of the indicated genotypes at E15.5 (**a, b**), P1 (**c, d**), and P7 (**e**). Ki67^{+} or PCNA-bright Sertoli cells are shown with yellow arrows as indicated right to the panels. Regions marked by yellow squares are magnified in the right panels. Bars in the large panels: 50 μm. Bars in the magnified panels: 20 μm.

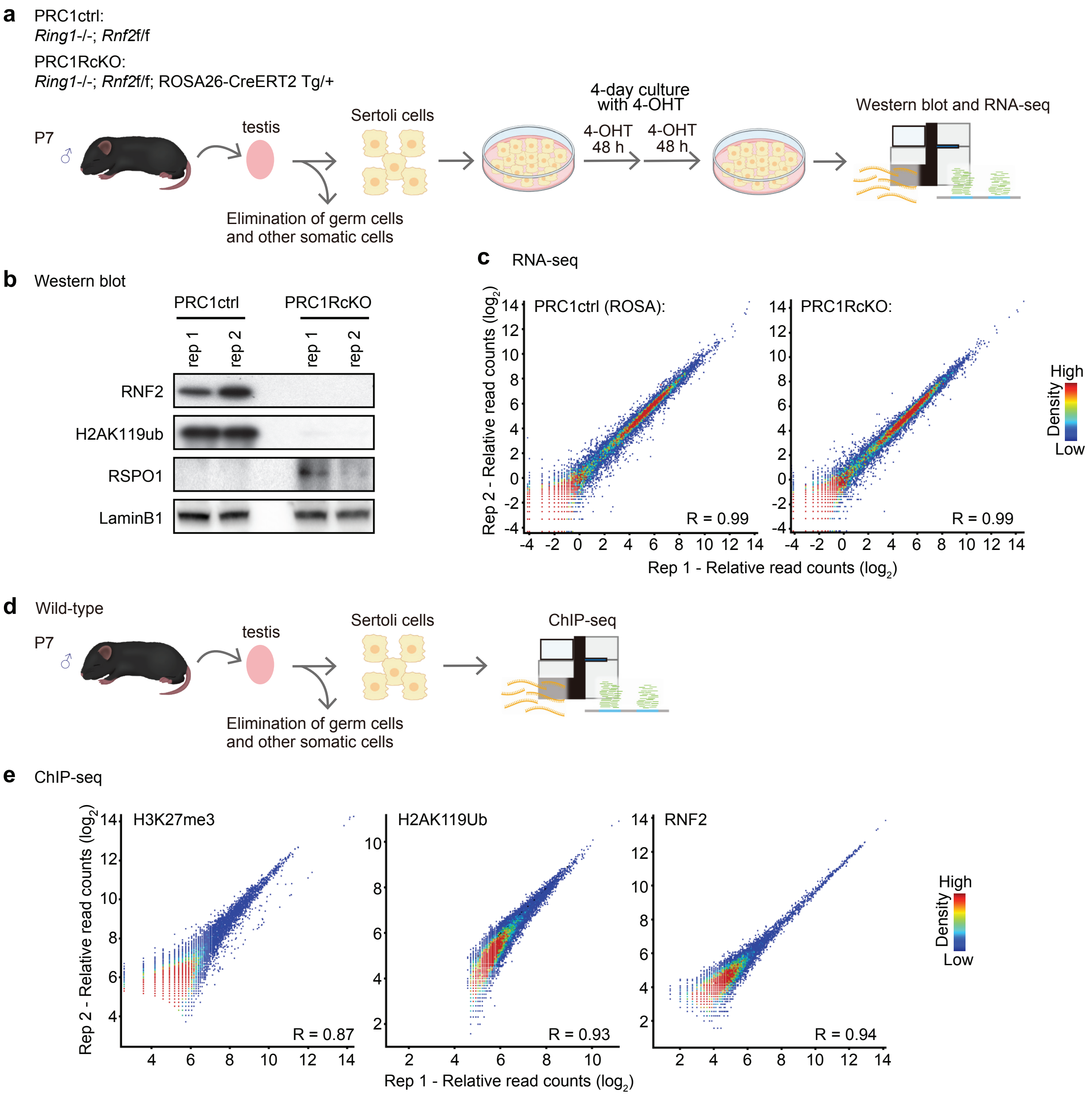

Supplementary Fig. 6. Confirmation of PRC1 deletion and biological replicates for RNA-seq and ChIP-seq data.
(**a**) Schematic of the experimental steps of tamoxifen-inducible depletion of PRC1 and Western blot and RNA-seq analyses.
(**b**) Western blots showing nearly complete depletion of RNF2 and H2A119ub in PRC1RcKO Sertoli cells. Ectopic expression of RSPO1 was observed, and Lamine B1 was used for loading control.
(**c**) Schematic of the experimental steps of ChIP-seq analysis.
(**d**) Scatter plots show the reproducibility of RNA-seq enrichment at individual peaks between biological replicates.
(**e**) Scatter plots show the reproducibility of ChIP-seq enrichment at individual peaks between biological replicates. The color scale indicates RNA-seq or ChIP-seq peak density. Pearson correlation values (R) are shown.

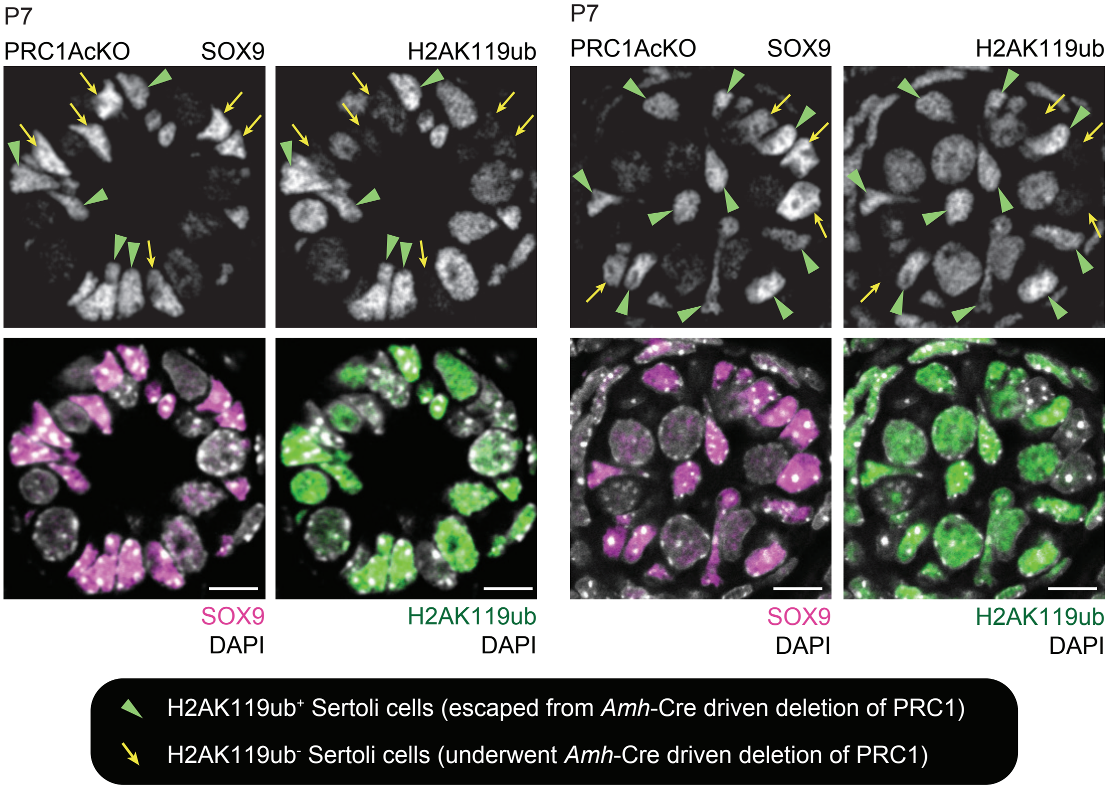

Supplementary Fig. 7. Expression of SOX9 in H2AK119ub-depleted Sertoli cells by *Amh*-Cre driven deletion.
Localization of SOX9 and H2AK119ub in PRC1AcKO at postnatal day 7 (P7). H2AK119ub^{+} Sertoli cells, which were escaped from *Amh*-Cre driven deletion of PRC1, are shown with green arrowheads. H2AK119ub^{-} Sertoli cells, which underwent *Amh*-Cre driven deletion of PRC1, are shown with yellow arrows. Two independent photos are showin. Bars: 10 µm.

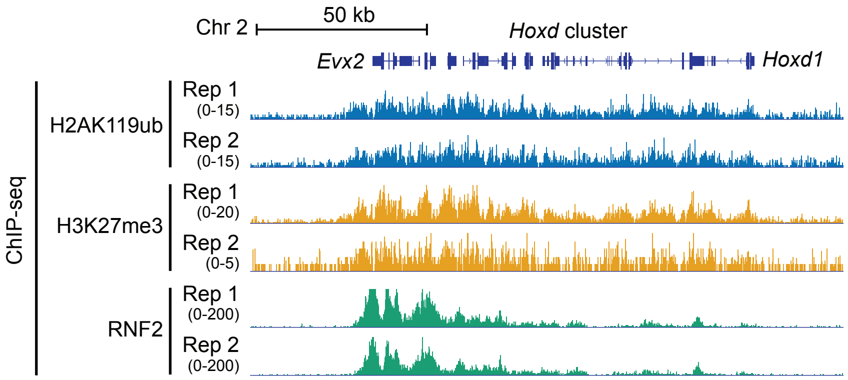

Supplementary Fig. 8. Track views of the *Hoxd* cluster.
Genome track views of the *Hoxd* cluster. ChIP-seq enrichment in wild-type Sertoli cells is shown. The numbers in brackets show the data ranges of normalized enrichment.

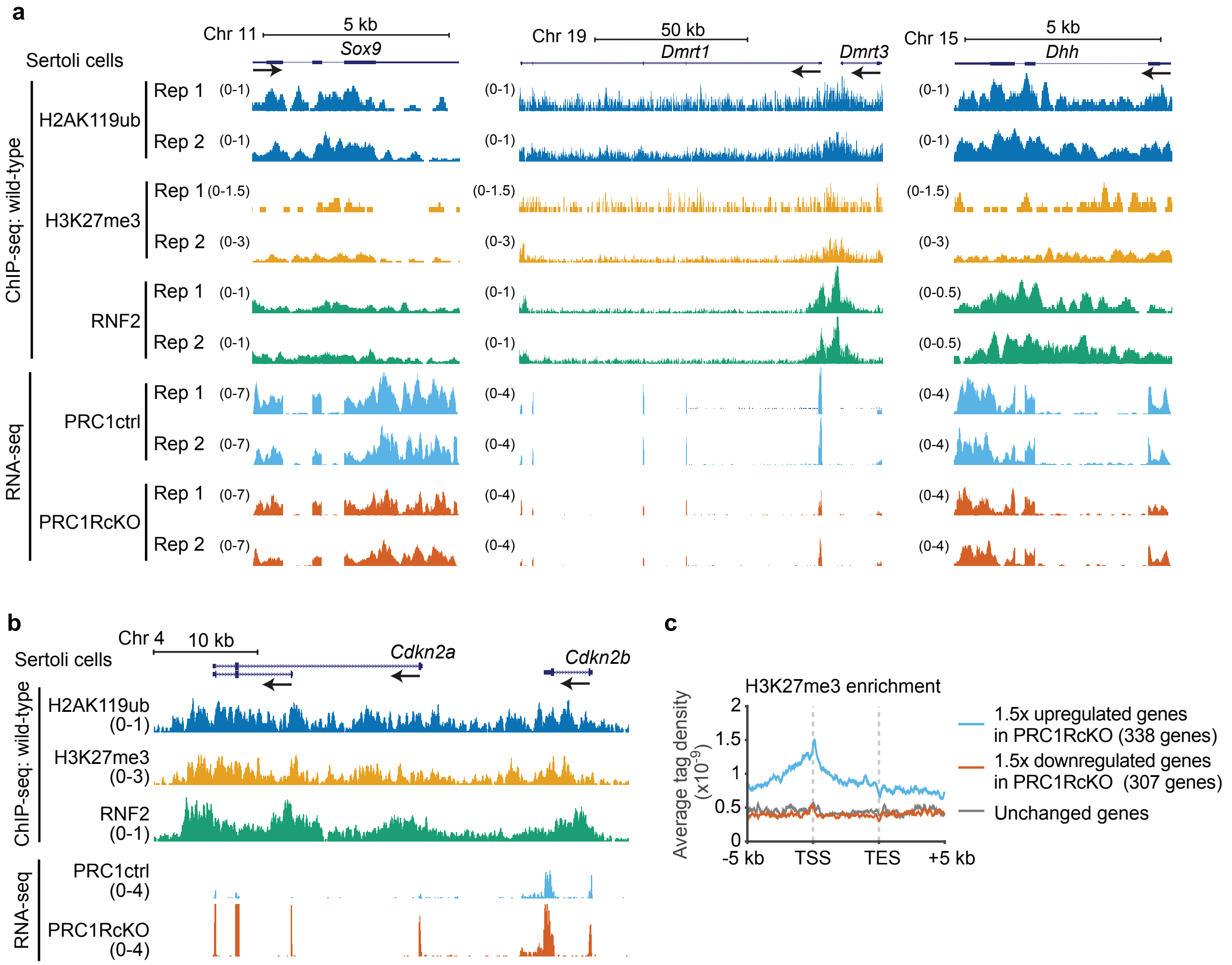

Supplementary Fig. 9. Genomic distribution of Polycomb marks.
(**a, b**) Genome track views of representative male genes (**A**) and the *Cdkn2* locus (**B**). ChIP-seq enrichment in wild-type Sertoli cells is shown (top); RNA-seq peaks in PRC1ctrl and PRC1RcKO Sertoli cells are shown (bottom). Arrows indicate the locations and directions of TSSs. The numbers in brackets show the data ranges of normalized enrichment.
(**c**) Average tag densities of H3K27me3 ChIP-seq enrichment on the groups of genes indicated in the panel.

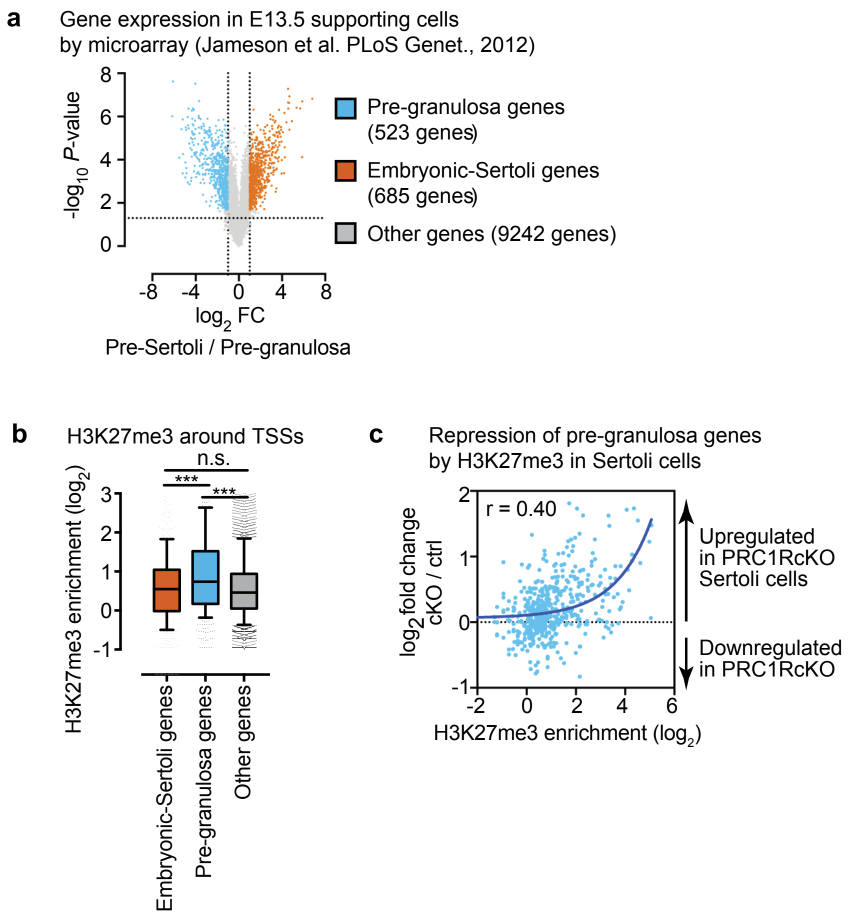

Supplementary Fig. 10. Expression profiles of specifically expressed genes in E13.5 granulosa cells and H3K27 enrichment.

(**a**) Microarray analysis of gene expression in E13.5 supporting cells (Jameson et al., 2012). Genes with the criteria of 2-fold higher expression in E13.5 XX supporting cells and $P < 0.05$ were termed as "Pre-granulosa genes" and shown in light blue. Genes with the criteria of 2-fold higher expression in E13.5 XY supporting cells and $P < 0.05$ were termed as "Embryonic-Sertoli genes" and shown in red.

(**b**) Box-and-whisker plots showing distributions of enrichment for H3K27me3 ChIP-seq data. Central bars represent medians, the boxes encompass 50% of the data points, and the whiskers indicate 90% of the data points. *** $P < 0.0001$, Mann-Whitney U tests.

(**c**) Scatter plots showing the correlation between ChIP-seq enrichment (±2 kb around TSSs) and gene expression in Sertoli cells. A Pearson correlation value (r) is shown in the panel. A linear trendline is shown in blue.

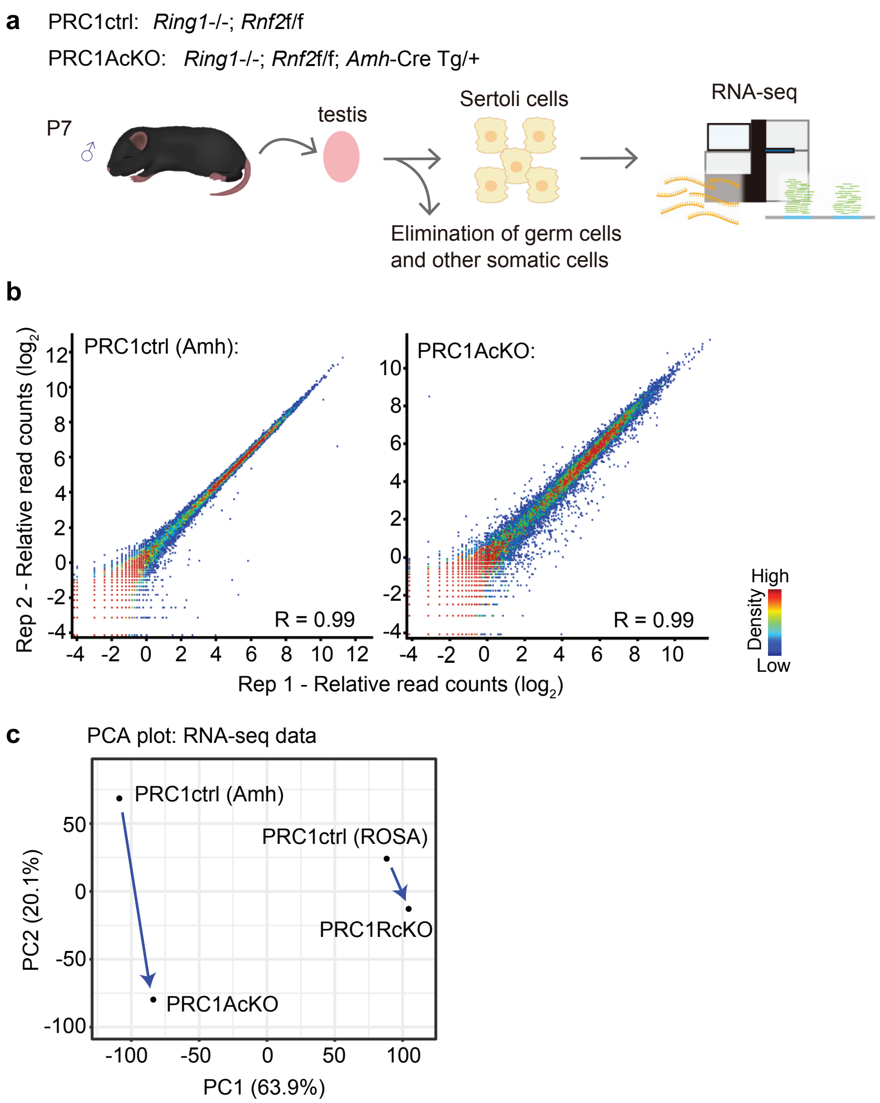

Supplementaly Fig. 11. Biological replicates for RNA-seq using PRC1AcKO cells

(**a**) Schematic of the experimental steps of RNA-seq analyses of PRC1AcKO Sertoli cells.

(**b**) Scatter plots show the reproducibility of RNA-seq enrichment at individual peaks between biological replicates. The color scale indicates RNA-seq or ChIP-seq peak density. Pearson correlation values (R) are shown.

(**c**) Principal component analysis of all RNA-seq data generated in this study.
