# Supplementary figures and images for: PRC1 suppresses a female gene regulatory network to ensure testicular differentiation
Source: Cell Death Dis. 2023 Aug 4;14(8):501. doi: 10.1038/s41419-023-05996-6 (PMC10403552; doi:10.1038/s41419-023-05996-6)

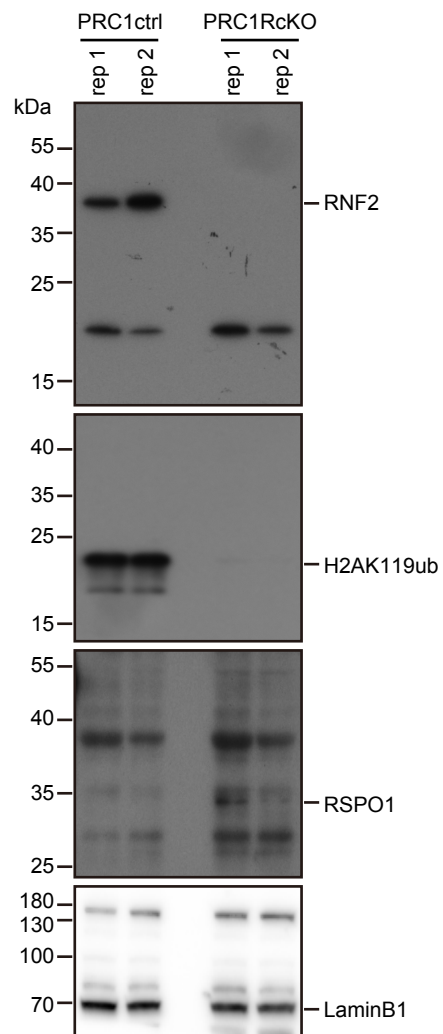

**Supplementary Material: the original western blots for Supplementary Fig. 6b**

Supplement: Supplementary file 8 — SupplementaryMaterial [file 41419_2023_5996_MOESM8_ESM.pdf]
